# Non-linear associations of cardiometabolic index with insulin resistance, impaired fasting glucose, and type 2 diabetes among US adults: a cross-sectional study

**DOI:** 10.3389/fendo.2024.1341828

**Published:** 2024-02-12

**Authors:** Jimei Song, Yimei Li, Junxia Zhu, Jian Liang, Shan Xue, Zhangzhi Zhu

**Affiliations:** ^1^ The First School of Clinical Medicine, Guangzhou University of Chinese Medicine, Guangzhou, China; ^2^ Department of Endocrinology, The First Affiliated Hospital of Guangzhou University of Chinese Medicine, Guangzhou, China

**Keywords:** cardiometabolic index, insulin resistance, impaired fasting glucose, type 2 diabetes, population-based study, NHANES

## Abstract

**Background:**

Cardiometabolic index (CMI) is a novel indicator for predicting the risk of obesity-related diseases. We aimed to determine the relationships of CMI with insulin resistance (IR), impaired fasting glucose (IFG), and type 2 diabetes mellitus (T2DM) using NHANES data from 1999 to 2020.

**Methods:**

After CMI values were estimated, weighted univariate and multivariate logistic regression analyses were used to ascertain whether CMI was an independent risk indicator for IR, IFG, and T2DM. Furthermore, stratified analyses and interaction analyses were carried out to investigate the heterogeneity of correlations across various subgroups. Subsequently, restricted cubic splines (RCS) were used to examine nonlinear relationships.

**Results:**

21,304 US adults were enrolled in our study, of whom 5,326 (22.38%) had IR, 4,706 (20.17%) had IFG, and 3,724 (13.02%) had T2DM. In the studied population, a higher CMI index value was significantly associated with an elevated likelihood of IR, IFG, and T2DM. In the RCS regression model, the relationship between CMI and IR, IFG, and T2DM was identified as nonlinear. A nonlinear inverted U-shaped relationship was found between CMI and IFG, and an inverse L-shaped association was observed between CMI and IR, CMI and T2DM. The cut-off values of CMI were 1.35, 1.48, and 1.30 for IR, IFG, and T2DM, respectively.

**Conclusion:**

Our results indicate that CMI was positively correlated with an increase in IR, IFG, and T2DM in the studied population. CMI may be a simple and effective surrogate indicator of IR, IFG, and T2DM.

## 1 Introduction

Insulin resistance (IR), due to a blunted response to insulin activity, is closely associated with the development and pathogenesis of metabolic dyshomeostasis, including impaired fasting glucose (IFG) and type 2 diabetes mellitus (T2DM) (1, 2). The prevalence of diabetes and prediabetes significantly increased to 36.5% in 2017–2020, which placed an enormous burden on public health and economies on a global scale (3). Epidemiological and pathophysiological studies have provided ample evidence that disproportionate body fat distribution and related abnormal lipid metabolism are intimately associated with the occurrence and deterioration of metabolic syndrome, diabetes, and IR (4). Furthermore, accurate metabolic measurements are crucial for assessing the severity of metabolic disorders and precluding irreversible metabolic conditions.

Anthropometry is a widely accepted and cost-effective tool for assessing an individual’s risk of adiposity, diabetes and metabolic syndrome. Recently, some traditional anthropometric indicators have been extensively utilized to quantify the accumulation of adiposity, such as triglyceride-glucose index (TyG), body mass index (BMI), waist-to-height ratio (WHtR) (5–7). Nevertheless, these indices fail to clearly differentiate fat distribution and provide evidence strong enough to be recognized as a premier marker of IR, IFG, and T2DM with excellent sensitivity or specificity (8, 9). Considering these factors, developing an accurate, cost-efficient and simple clinically compatible alternative approach is urgently needed.

More recently, as a result of its ease of use and affordability, the Cardiometabolic index (CMI) was originally introduced by Ichiro Wakabayashi in 2015, which is computed as a product of WHtR and triglyceride-to-HDL cholesterol ratio (TG/HDL ratio) (10). Owing to its integrated abdominal obesity and dyslipidemia indices, CMI was validated to be a robust and independent clinical adiposity discriminator as well as a closer correlation with metabolic abnormality than traditional anthropometric indicators (11, 12). Additionally, prior CMI-related studies have demonstrated that CMI can detect the presence of obesity-related disorders and cardiovascular dysfunction, including diabetes, hyperuricemia, non-alcoholic fatty liver disease, hypertension, stroke, kidney disease, erectile dysfunction, and peripheral arterial disease (10, 13–20).

Previous studies in Chinese and Japanese populations have demonstrated that CMI is a predictor of diabetes (21, 22). However, sparse comprehensive studies are available to ascertain the capability of CMI in identifying individuals with IR and T2DM, especially with IFG, in US adults. Consequently, to address these knowledge gaps, this research was conducted to systematically investigate the associations between CMI and IR, IFG, and T2DM in a sizable cohort of individuals.

## 2 Methods

### 2.1 Data source and study sample

The National Health and Nutrition Examination Survey (NHANES) is a nationally representative cross-sectional survey of the United States that employs a multistage, stratified, subgroup random sample study design. In addition to standardized in-home interviews, the survey covers a wide range of aspects, such as health examinations and laboratory tests, with all participants providing written informed consent. Detailed statistics are accessible on its website (https://www.cdc.gov/nchs/nhanes/).

Our investigation included the analysis of CMI in relation to three separate dependent variables: IR, IFG, and T2DM. To expand the sample size and ensure that the data was comprehensive, the data utilized in our study were obtained from the NHANES database for 10 periods (1999–2020, involving 107,622 participants).

**Figure 1:**
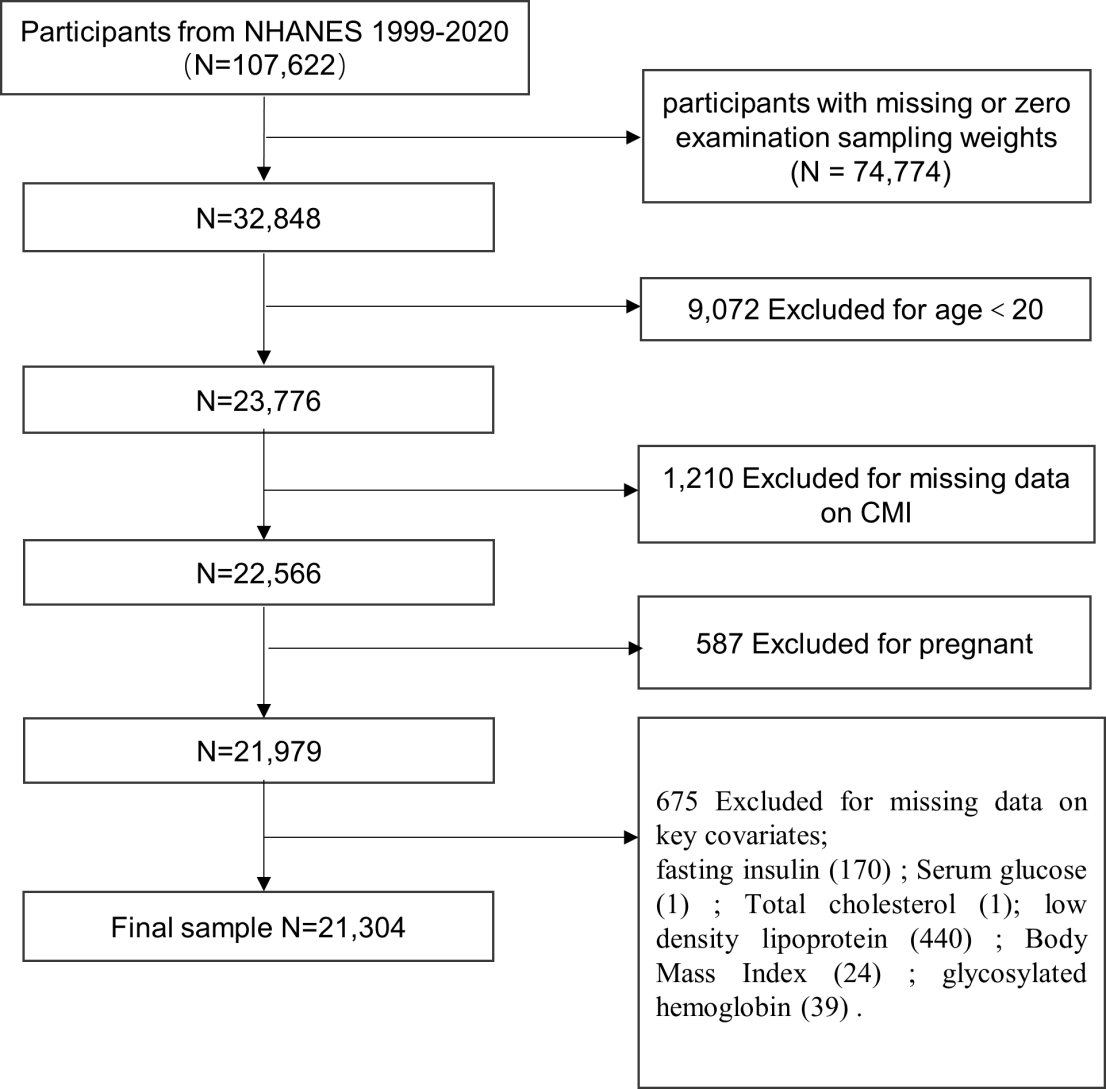
Flow chart of study sample screening.

We excluded participants with missing sampling fasting weights or sampling fasting weights equal to zero (n = 74,774). In addition, 20 years or younger participants were excluded from further analysis (n = 9,072). We also excluded the ones with missing data on CMI (n = 1,210), who were pregnant (n = 587), and those missing data on key covariates (n = 675), namely, fasting insulin (FSI), fasting plasma glucose (FPG), BMI, total cholesterol (TC), low-density lipoprotein (LDL), glycosylated hemoglobin (HbAlc). Ultimately, 21,304 participants (weighted n = 201,348,819) were enrolled in the final analytic cohort (**Figure 1**).

### 2.2 Exposure variable and outcome variables

The CMI was the exposure variable, which was mathematically calculated using the following formula: TG (mmol/L)/HDL (mmol/L) * [WC (cm)/HT (cm)]. Furthermore, according to the CMI quartiles, participants were subdivided into four groups. More specifically, the first quartile (CMIQ1) served as the reference, including quartile1 (CMIQ1 ≤ 25th percentile, 0.03-0.30), quartile2 (CMIQ2 > 25th percentile to ≤ 50th percentile, 0.30-0.51), quartile3 (CMIQ3 > 50th percentile to ≤ 75th percentile, 0.51-0.90), and quartile4 (CMIQ4 > 75th percentile, 0.90-13.73).

The outcome variables included in this study were the incidence of IR, IFG, and T2DM. Referring to other pertinent studies, IR was characterized by the homeostasis model assessment for insulin resistance (HOMA-IR) values equal to or greater than the 75th percentile (HOMA-IR > 3.94). HOMA-IR was calculated using the following equation: [FPG (mmol/L) * FSI (µU/ml)]/22.5 (23). IFG was characterized by FPG ≥ 5.6 mmol/L and < 7.0 mmol/L as per the American Diabetes Association (ADA) guidelines 2023 criteria (24). T2DM was defined as FPG ≥ 7.0 mmol/L, 2-h oral glucose tolerance test plasma glucose ≥ 11.1 mmol/L, HbA1c ≥ 6.5%, the use of hypoglycemic medications, or individuals who self-reported having diabetes.

### 2.3 Covariates

Covariates were decided based on the already known confounding factors from clinical plausibility and previous studies. More precisely, demographic factors included age, sex, race/ethnicity, levels of education, marital status, and poverty income ratio (PIR). Questionnaires about alcohol consumption and sedentary behavior were also included. Additionally, laboratory parameters such as serum cotinine levels, blood urea nitrogen (BUN), serum creatinine (Scr), FPG, FSI, TC, LDL, HDL, HbA1c, estimated glomerular filtration rate (eGFR), and HOMA-IR were evaluated. Anthropometric indicators included body weight (WT), WC, and BMI. Hypertension, cardiovascular disease (CVD), dyslipidemia, chronic kidney disease (CKD), and cancer were recognized as significant susceptibility indicators for IR, IFG, and T2DM. Further detailed information about each variable are available on the NHANES website.

In this study, age was subdivided into three categories: 20–39, 40–59, and 60 years or older. Race was designated as Mexican American, non-Hispanic White, non-Hispanic Black, other Hispanic, and other races. Three ordinal groups were used to categorize educational attainment: less than high school, high school or equivalent, and college or above. In addition, PIR was used to measure the level of income and was divided into three categories: < 1.3, 1.3–1.8, and > 1.8. Marital status were categorized as widowed, married, separated, divorced, living with their partners, never married, and others. Furthermore, there were three subdivided alcohol consumption categories: none, moderate (> 0 to ≤ 1 drink/d for women or > 0 to ≤ 2 drinks/d for men), heavy (> 1 drink/d for women or > 2 drinks/d for men). Smoking status was categorized based on the serum cotinine levels into low level (< 0.015 ng/mL), moderate level (0.015–3 ng/mL), and high level (> 3 ng/mL). The sedentary behavior was evaluated using a self-administered physical activity questionnaire (PAQ) and confirmed if the participant’s physical activity level was neither vigorous nor moderate in the preceding month (PAD200 or PAD320) (25). Furthermore, BMI was stratified into underweight (< 18.5 kg/m^2^), normal weight (18.5–25.0 kg/m^2^), overweight (25.0–29.9 kg/m^2^), and obesity (≥ 30.0 kg/m^2^). The Chronic Kidney Disease Epidemiology Collaboration equation was used to estimate eGFR (26). Furthermore, eGFR values < 60 mL/min/1.73 m^2^ were established as the diagnostic threshold of CKD. Moreover, laboratory-measured levels of TC, TG, HDL, LDL, INS, BUN, Scr, and HOMA-IR were evaluated.

Hypertension was considered present if at least one of the following criteria was satisfied: (1) average diastolic blood pressure ≥ 80 mmHg, (2) average systolic blood pressure ≥ 130 mmHg, (3) the use of anti-hypertensive medication, or (4) individuals who self-reported having hypertension. Dyslipidemia was screened by the following criteria: (1) TG ≥ 1.7 mmol/L, (2)TC ≥ 5.18 mmol/L, (3) LDL ≥ 3.37 mmol/L, (4) HDL < 1.04 mmol/L for males and < 1.3 mmol/L for females, or (5) the use of cholesterol-lowering drugs. The history of CVD encompassed a composite event, including coronary heart disease, congestive heart failure, heart attack, angina, and stroke.

### 2.4 Statistical analysis

Weighted analyses (WTSAFPRP) were carried out based on the complex sampling survey in accordance with the NHANES recommendations. Continuous variables were presented herein as weighted mean ± standard errors (SE), and the independent samples t–test and Mann–Whitney test were utilized to compare the two groups. Categorical variables were represented as weighted percentages with a 95% confidence interval (CI) and compared using the weighted chi-square test. Weighted univariate and multivariate logistic regression analyses were carried out to evaluate the robustness of the correlations between CMI and IR, IFG, and T2DM in the three different models. Confounding variables were not adjusted in Model 1. Sex, age, and race were adjusted in Model 2. Finally, age, gender, race, marital status, education level, sedentary behavior, alcohol use, BMI, serum cotinine levels, and the PIR, BUN, Scr, TC, LDL, WT, eGFR, BMI, hypertension, and CVD were adjusted in Model 3. Missing values were filled in using the mode of categorical variables.

Subsequently, to investigate the heterogeneity of interrelations across various subgroups, stratified analyses and interaction analyses were performed to investigate whether the causality varied based on some socioeconomic and life behavior characteristics, including age, sex, race, education level, marital status, PIR, smoking status, alcohol consumption, sedentary behavior, BMI, high level of TC and LDL, dyslipidemia, hypertension, CVD, cancer, eGFR, and CKD. If the interaction P-value < 0.05, the results were considered significant and reliable. Otherwise, the results indicated the existence of a peculiar population. Additionally, adjusted restricted cubic spline (RCS) regression analysis was used to determine the non-linearity of the relationships of CMI with IR, IFG, and T2DM. Non-linearity was tested using the likelihood ratio test. P_overall_ < 0.05 and P_non – linearity_ < 0.05 conditions indicated a nonlinear dose–response relationship.

All statistical analyses were executed using R (version 4.2.2, http://www.R-project.org) and EmpowerStats (version 3.4.3, www.empowerstats.com), with a two-sided P-value < 0.05 deemed statistically significant.

## 3 Results

### 3.1 Characteristics of the participants

After a rigorous screening process, 21,304 participants were included in our study. Among them, 51.3% were women, the mean age was 47.05 ± 0.15 years, and non-Hispanic Whites constituted the largest proportion (68.01%). Importantly, participants with high CMI values had higher incidences of IR, IFG, and T2DM. The overall prevalence of IR was 22.38% (95% CI: 21.65–23.13), which was 3.53% (95% CI: 3.01–4.13), 13.05% (95% CI: 11.87–14.33), 26.29% (95% CI: 24.78–27.87), and 48.44% (95% CI: 46.62–50.26) in CMIQ1, CMIQ2, CMIQ3, and CMIQ4, respectively (P < 0.01). The prevalence of IFG was 20.17% (95% CI: 19.46–20.9), which was 10.17% (95% CI: 9.18–11.25), 17.36% (95% CI: 16.06–18.74), 23.33% (95% CI: 21.86–24.87), and 30.74% (95% CI: 29.04–32.49) in CMIQ1, CMIQ2, CMIQ3, and CMIQ4, respectively (P < 0.01). The morbidity of T2DM was 13.02% (95% CI: 12.47–13.59), which was 4.39% (95% CI: 3.84–5.01), 9.08% (95% CI: 8.22–10.02), 14.86% (95% CI: 13.73–16.07), and 24.54% (95% CI: 23.04–26.11) in CMIQ1, CMIQ2, CMIQ3, and CMIQ4, respectively (P < 0.01).

Notably, participants with higher CMI tended to be males [37.62% (95% CI: 35.91–39.37) in CMIQ1 vs. 59.34% (95% CI: 57.56–61.1) in CMIQ4], with older ages, higher smoking status, higher education levels, more non-drinkers, more overweight or obese, married, with lower income, and more likely to have lower levels of HDL, higher levels of BMI, WC, WT, FPG, HbA1c, INS, HOMA-IR, TC, TG, LDL, BUN, and Scr (all P < 0.05), with higher prevalence of hypertension, dyslipidemia, CVD, CKD, and cancer. **Supplementary Table 1** displays the comprehensive baseline characteristics of the included individuals.

### 3.2 The association of CMI with IR, IFG, and T2DM

The logistic regression analysis results for the relationship between CMI and IR, IFG, and T2DM in both continuous and categorical analyses are presented in **Table 1**. Three models (Models 1, 2, and 3) were constructed to analyze the independent association of CMI with IR, IFG, and T2DM.

**Table 1:** Associations between CMI with IR,IFG and T2DM.

IR	Events yes	model1	model2	model3
		OR (95% CI) p-Value	OR (95% CI) p-Value	OR (96% CI) p-Value
CMI	5,326	5.06(4.6,5.57) <0.01	5.29(4.78,5.84) <0.01	3.46(3.1,3.85) <0.01
CMIQ1	250	Reference	Reference	Reference
CMIQ2	804	4.11(3.37,5) <0.01	4.17(3.42,5.08) <0.01	2.37(1.9,2.94) <0.01
CMIQ3	1,576	9.76(8.13,11.71) <0.01	10.15(8.42,12.23) <0.01	4.33(3.52,5.33) <0.01
CMIQ4	2,696	25.7(21.48,30.75) <0.01	28.25(23.44,34.05) <0.01	9.87(8.01,12.17) <0.01
P for trend		P<0.01	P<0.01	P<0.01
IFG				
CMI	4,706	1.83(1.7,1.97) <0.01	1.7(1.57,1.84) <0.01	1.27(1.16,1.4) <0.01
CMIQ1	631	Reference	Reference	Reference
CMIQ2	1,046	1.86(1.6,2.15) <0.01	1.59(1.37,1.85) <0.01	1.23(1.06,1.44)0.01
CMIQ3	1,359	2.69(2.34,3.1) <0.01	2.17(1.88,2.51) <0.01	1.42(1.22,1.67) <0.01
CMIQ4	1,670	3.92(3.41,4.51) <0.01	3.15(2.72,3.65) <0.01	1.77(1.49,2.1) <0.01
P for trend		P<0.01	P<0.01	P<0.01
T2DM				
CMI	3,724	2.44(2.27,2.63) <0.01	2.69(2.47,2.92) <0.01	2.01(1.82,2.22) <0.01
CMIQ1	365	Reference	Reference	Reference
CMIQ2	712	2.17(1.82,2.59) <0.01	1.9(1.58,2.28) <0.01	1.59(1.3,1.94) <0.01
CMIQ3	1,091	3.8(3.22,4.49) <0.01	3.25(2.72,3.87) <0.01	2.35(1.92,2.87) <0.01
CMIQ4	1,556	7.09(6.03,8.33) <0.01	6.94(5.81,8.28) <0.01	4.08(3.34,4.98) <0.01
P for trend		P<0.01	P<0.01	P<0.01

Model 1: Non-adjusted model; Model 2 adjusted for: gender; age; race; Model 3 adjusted for: age, gender, race, marital status, level of education, alcohol use, BMI, sedentary behavior, serum cotinine levels, and the PIR, BUN, SCR, TC, LDL, WT, eGFR, BMI, hypertension, CVD.

As illustrated by the continuous analysis, CMI was positively correlated with the prevalence of IR, IFG, and T2DM in all models (P for trend < 0.01). In our fully adjusted model (Model 3), the association between CMI and IR was positive (odds ratio [OR] = 3.46, 95% CI: 3.1–3.85), and so was the association of CMI with IFG (OR = 1.27, 95% CI: 1.16–1.4) and with T2DM (OR = 2.01, 95% CI: 1.82–2.22).

When we grouped CMI according to quartiles, regardless of adjustment for covariates, the categorical analysis demonstrated that this noticeable correlation persisted. With the escalation of CMI, the OR values for IR, IFG, and T2DM increased as well (P for trend < 0.01). Meanwhile, in Model 3, CMIQ1 was used as a reference group. In the group of CMIQ4 (0.90, 13.73), the association between CMI and IR was positive (OR = 9.87, 95% CI: 8.01–12.17), as was the association of CMI with IFG (OR = 1.77, 95% CI: 1.49–2.10). Meanwhile, the association between CMI and T2DM was also positive (OR = 4.08, 95% CI: 3.34–4.98), with all P-values for trend < 0.01, revealing that subjects with higher CMI were at a greater risk of IR, IFG, and T2DM.

### 3.3 Results of subgroup analysis

Subgroup analysis and interaction analysis for the association of CMI with IR, IFG, and T2DM were performed based on age, sex, race, education level, marital status, PIR, BMI, eGFR, smoking status, alcohol consumption, sedentary behavior, history of CVD, cancer, dyslipidemia, hypertension.

**Figure 2:**
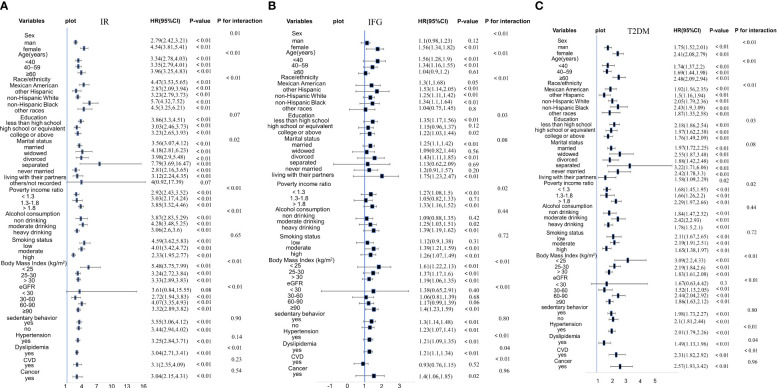
**(A)** Stratified analyses and interaction analyses of the association of CMI and IR. **(B)** Stratified analyses and interaction analyses of the association of CMI and IFG. **(C)** Stratified analyses and interaction analyses of the association of CMI and T2DM.

The results of the subgroup analysis (**Figure 2**) indicated that the results of our study are rather stable. For participants with IR, statistically significant interactions were observed between CMI and age, sex, race, marital status, PIR, alcohol consumption, BMI, eGFR, and dyslipidemia (P for interaction < 0.05). For participants with IFG, statistically significant connections were observed between CMI and age, sex, race, PIR, BMI, eGFR, dyslipidemia, hypertension, CVD, education level (all P for interaction < 0.05). For individuals with T2DM, significant relationships were found between CMI and age, sex, race, BMI, eGFR, dyslipidemia, cancer, CVD, and alcohol consumption (all P for interaction < 0.05).

The positive association of CMI with IR, IFG, and T2DM was stable across all genders. However, the correlation was more pronounced in female participants than in male participants (females OR = 4.54, males OR = 2.79 in IR participants; females OR = 1.56, males OR = 1.1 in IFG participants; females OR = 2.41, males OR = 1.75 in T2DM participants). However, the CMI of male participants was not related to IFG participants in Model 3 (P = 0.12). Besides, although the positive associations of CMI with IR and T2DM remained stable across all age groups, IFG participants aged ≥ 60 years had no relationship with CMI (P = 0.61). Furthermore, the positive association of CMI with IR and T2DM remained stable across all races (P < 0.05). We observed that the positive associations of CMI with IR and T2DM were more significant in non-Hispanic Black and that between CMI and IFG was more significant in other Hispanics.

### 3.4 Nonlinear associations between CMI and the prevalence of IR, IFG, and T2DM

**Figure 3:**
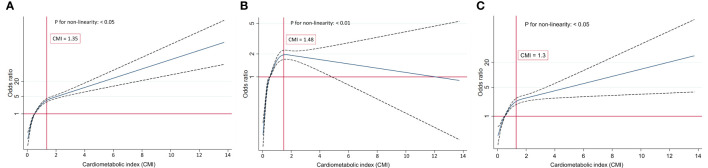
**(A)** Restricted cubic splines of CMI and IR. **(B)** Restricted cubic splines of CMI and IFG. **(C)** Restricted cubic splines of CMI and T2DM.

RCS regression with multivariable-adjusted associations was adopted to visually demonstrate dose–response relationships between CMI and the prevalence of IR, IFG, and T2DM. We used the median value of CMI (0.51) as the reference point and observed nonlinear relationships of CMI with IR, IFG, and T2DM (**Figure 3**; P < 0.01).

The analysis of the link between CMI and the risk of IFG using the RCS plot demonstrated a nonlinear inverted U-shaped relationship (P for nonlinearity < 0.01). The participants’ risk of IFG gradually increased at the beginning and then decreased once the turning point of CMI was reached (CMI = 1.48). When CMI ≤ 1.48, the increasing prevalence of IFG was positively correlated with the increasing CMI. Conversely, when CMI > 1.48, the risk of IFG dampened with the increase of CMI. The relationships of CMI with IR and T2DM were observed to be inversely L-shaped (all P for nonlinearity < 0.05). The risk of IR and T2DM boosted rapidly with the increase in CMI before reaching the turning point (CMI = 1.35 in IR, CMI = 1.30 in T2DM). However, the trend for the two flattened out when the CMI value was greater than 1.35 and 1.30, respectively.

## 4 Discussion

As a widespread metabolic disorder with high prevalence, IR is a risk factor and a precursor of cardiovascular illnesses and diabetes. IFG, an intermediate stage that precedes diabetes and increases the likelihood of developing diabetes, was recognized by the American Diabetes Association in 1997 (27). Direct techniques such as the hyperinsulinemic euglycemic clamp or the insulin suppression test are recognized as the gold standard methods for testing IR (28). Nevertheless, their application is restricted in large clinical and epidemiological studies as they are expensive, invasive, and complicated. Consequently, a simpler metric and more convenient metabolic-related determination system is essential for clinical purposes.

Our investigation helped us demonstrate strong, positive, substantial evidence to corroborate the speculation that CMI was linked to the incidence of IR, IFG, and T2DM in US adults regardless of whether CMI are modeled as continuous or categorical variables or whether major confounding variables were adjusted for (P for interaction < 0.05). Most importantly, our study supported that CMI has a deteriorated metabolic profile, and it can function as a reproducible screening marker of IR, IFG, and T2DM. We propose it as an economical approach for early diabetic management. Although the logistic regression analysis demonstrated that the associations between CMI and IR, IFG, T2DM are significant, we can see the relationship between CMI and IFG appears to be weaker than the relationship between IR and T2DM. It possibly restricted the accuracy and specificity of CMI to IFG for the clinical diagnosis. Therefore, additional researches on other indicators that are more sensitive to IFG are warranted.

We further validated the associations of CMI with IR, IFG, and T2DM in subgroups and found that the results were stable except for gender and age in IFG (P < 0.05). Specifically, stronger relationships were observed in the CMIQ4 groups. Notably, there were gender disparities that females were found to have a higher risk of IR, IFG, and T2DM than males in our study, and it appears to have been found consistent with several prior studies in Chinese and Japanese populations (21, 22). Sex distributions could be related to various mechanisms, including fat micro-environment, cell-intrinsic characteristics, and sex hormones (29). An increase in female hormones, especially estrogen, modulates lipolysis and lipogenesis via estrogen receptor-α and plays a significant role in adipose tissue expansion and remodeling during IR, IFG, and T2DM progression (30).

Our interaction analyses suggested that statistically significant connections were observed between CMI and eGFR, which is in accordance with Manti Miao et al.’s observation that CMI was independently associated with microalbuminuria and renal function, particularly in diabetic individuals (31). Collectively, an inverse L-shaped association was observed between CMI and IR, T2DM, respectively. while an inverse U-shaped association was observed between CMI and IFG. There was an increasing trend in IR, IFG, and T2DM occurrence with increasing CMI. Moreover, the trend gradually plateaued in participants with IR and T2DM compared with the left side of the inflection point (CMI = 1.35 in IR; CMI = 1.30 in T2DM). Intriguingly, when CMI > 1.48, the trend gradually declined in IFG participants.

Several previous CMI-related studies have predominantly focused on Japanese and Chinese, and have demonstrated the excellent predictive capability of CMI. This index is deemed a simple, easily calculable, as well as cost-effective anthropometric indicator. Our current study’s findings are consistent with some previous CMI-related studies. In a Japan-based cohort study, Fubing Zha et al. demonstrated that CMI level had a nonlinear association with the incidence of diabetes (21). Similar findings were also reported in another study conducted in middle-aged and older Chinese (22). Wakabayashi et al. retrospectively analyzed 1,411 Japanese males with diabetes and confirmed an inverse association between alcohol drinking and CMI (32). Meanwhile, Lazzer et al. demonstrated that CMI (AUC = 0.86) could serve as an alternative for predicting metabolic syndrome measurement, and the cut-off point was CMI > 0.84 (33). Consistently, a study in the general Chinese population demonstrated that an increasing CMI was linked to a higher odds of diabetes, and the ROC results revealed a strong discriminating power of CMI (34). Based on these results as well as our observation, we noted the detrimental impact of patients with high CMI on the incidence of metabolic disorders. However, Acosta-García et al. assessed CMI’s predictive power for changes in fasting glucose levels, dyslipidemia, and hypertension in adolescents (35). CMI demonstrated the capacity to forecast hypertension and dyslipidemia but not IFG. The area under the ROC curve of IFG was 0.564 (95% CI: 0.447 to 0.682). The results obtained are not consistent with our findings. The discrepancies could be partly ascribed to racial differences, the included population of Acosta-García’s study was from Venezuela. Besides, it may also be explained by the fact that study’s included covariates are different.

The precise mechanism underlying the associations of CMI with IR, IFG, and T2DM requires further exploration. Excess adipose tissue may promote IR by altering the inflammatory micro-environment, boosting the production of resistin or pro-inflammatory cytokines, such as tumor necrosis factor-α and interleukin-6, which can trigger low-grade chronic inflammatory response and oxidative stress (36, 37). Furthermore, endoplasmic reticulum stress, innate immunological responses, mitochondrial dysfunction, and lipotoxicity may also be significant for fat accumulation leading to IR, IFG, and T2DM. In summary, inflammation and lipid metabolism-related disorders are vital mechanisms in progressing IR, IFG, and T2DM.

CMI is a comprehensive indicator of obesity-related disorders that integrates abdominal obesity and dyslipidemia indexes, all of which are pivotal metabolic disturbance drivers. Previous studies established that TG/HDL-C was strongly associated with metabolic disorders, IR, and obesity (38). High plasma TG levels decreased the number of insulin receptors on adipocytes, and prevented insulin from binding to insulin receptors, leading to diabetes. Decreased insulin secretion and sensitivity are also caused by low HDL-C levels (39). WC and WHtR have been utilized extensively in previous studies to evaluate the degree of abdominal adiposity and may exert a deleterious impact on β-cell function. Obese individuals with excess free fatty acid circulation and high level of WHtR can prevent glucose transport activity, limiting the contribution of insulin to glucose metabolism and resulting in IR (40). Furthermore, the reason underlying the association between CMI and the incidence of IR, IFG, and T2DM could be explained by CMI being an intuitive and trustworthy indicator of metabolic diseases.

## 5 Limitations and strengths of this study

The main strength of this study is that the data analyzed were acquired from the NHANES, a high-quality assurance database that has received international recognition and rigorous validation, which was a weighted design that is national and representative in scope. In addition, to accurately estimate the correlation, numerous potentially confounding covariates were evaluated and adjusted. Moreover, to enhance statistical power, we used stratified analysis and RCS to further analyze the association of CMI with IR, IFG, and DM.

This study also has a few limitations that merit acknowledged. First, our study was cross-sectional, which inevitably has some bias precluding us from determining temporal relationships and establishing causality. Therefore, further prospective studies are required to investigate the relationships. Second, although we have controlled and eliminated many confounding factors, we cannot entirely rule out the unmeasured or unidentified variables, such as dietary patterns, drug histories, and genetics. Third, we assessed IR using the HOMA-IR index, which is not the “gold standard” approach. However, HOMA-IR may be more appropriate in extensive epidemiological investigations across diverse populations (41). Fourth, the population observed in this study was only limited to American adults. The applicability of our study’s results to other racial populations may not be extrapolatable, which needs further research.

## 6 Conclusion

In conclusion, the results of our study indicated that CMI was an effective, straightforward, easily accessible, and low-cost reliable surrogate indicator of obesity-related disorders. Our study shed new light on a simpler way to evaluate individuals with IR, IFG, and T2DM in the US adult population. Furthermore, CMI may provide recommendations for clinical practice and public health, particularly in underdeveloped countries and ill-equipped laboratories. However, additional investigations are crucial to validate the underlying mechanisms based on our findings.

## Data availability statement

The datasets presented in this study can be found in online repositories. The names of the repository/repositories and accession number(s) can be found in the article/**Supplementary Material**.

## Ethics statement

The studies involving humans were approved by the National Center for Health Statistics (NCHS). The studies were conducted in accordance with the local legislation and institutional requirements. The participants provided their written informed consent to participate in this study.

## Author contributions

JS: Writing – original draft, Writing – review & editing, Data curation, Formal analysis, Investigation, Methodology, Software. YL: Formal analysis, Investigation, Methodology, Writing – review & editing. JZ: Formal analysis, Methodology, Writing – review & editing. JL: Methodology, Writing – original draft. SX: Methodology, Writing – review & editing. ZZ: Conceptualization, Funding acquisition, Supervision, Validation, Writing – review & editing.
