# Supplementary material for: Non-linear associations of cardiometabolic index with insulin resistance, impaired fasting glucose, and type 2 diabetes among US adults: a cross-sectional study
Source: Front Endocrinol (Lausanne). 2024 Feb 12;15:1341828. doi: 10.3389/fendo.2024.1341828 (PMC10894973; doi:10.3389/fendo.2024.1341828)
Supplement: Supplementary file 1 [file Table_1.docx]

Supplementary Material

# Supplementary Table 1

Table1 General characteristics of included participants (n = 21304) by quartile of CMI in NHANES 1999–2020.

| Characters | total | CMIQ1 | CMIQ2 | CMIQ3 | CMIQ4 | P value |
| --- | --- | --- | --- | --- | --- | --- |
| SEX |  |  |  |  |  | <0.01 |
| man | 48.7(47.8-49.61) | 37.62(35.91-39.37) | 47.34(45.52-49.17) | 51.48(49.68-53.28) | 59.34(57.56-61.1) |  |
| female | 51.3(50.39-52.2) | 62.38(60.63-64.09) | 52.66(50.83-54.48) | 48.52(46.72-50.32) | 40.66(38.9-42.44) |  |
| Age | 47.05±0.15 | 43.18±0.28 | 46.96±0.3 | 48.93±0.29 | 49.47±0.27 | <0.01 |
| <40 years | 37.32(36.45-38.2) | 47.65(45.88-49.42) | 37.78(36.03-39.57) | 33.16(31.48-34.9) | 29.76(28.13-31.43) |  |
| ≥60 years | 25.28(24.54-26.03) | 18.96(17.69-20.29) | 25.85(24.35-27.41) | 28.75(27.24-30.31) | 28.12(26.57-29.72) |  |
| Race/ethnicity |  |  |  |  |  | <0.01 |
| Mexican American | 8.16(7.83-8.5) | 5.56(5.04-6.14) | 7.72(7.1-8.39) | 9.32(8.64-10.06) | 10.27(9.53-11.05) |  |
| other Hispanic | 5.71(5.39-6.05) | 4.51(3.95-5.14) | 5.59(5-6.25) | 6.55(5.87-7.31) | 6.32(5.67-7.05) |  |
| non-Hispanic White | 68.01(67.29-68.73) | 66.96(65.51-68.38) | 67.44(65.97-68.88) | 66.59(65.08-68.06) | 71.13(69.72-72.5) |  |
| non-Hispanic Black | 10.97(10.6-11.35) | 15.48(14.61-16.39) | 12.54(11.75-13.36) | 9.85(9.15-10.6) | 5.58(5.08-6.13) |  |
| other races | 7.15(6.76-7.56) | 7.49(6.76-8.3) | 6.71(5.97-7.53) | 7.69(6.87-8.6) | 6.69(5.95-7.52) |  |
| Education |  |  |  |  |  | <0.01 |
| less than high school | 59.26(58.39-60.13) | 68.05(66.45-69.6) | 59.48(57.69-61.24) | 56.78(55.02-58.53) | 51.97(50.16-53.79) |  |
| High school or equivalent | 24.21(23.43-25) | 20.46(19.09-21.9) | 24.66(23.05-26.33) | 24.65(23.12-26.25) | 27.36(25.72-29.07) |  |
| college or above | 16.47(15.92-17.04) | 11.42(10.55-12.36) | 15.84(14.76-16.98) | 18.49(17.31-19.73) | 20.6(19.36-21.89) |  |
| not recorded | 0.06(0.03-0.11) | 0.07(0.02-0.28) | 0.03(0.01-0.13) | 0.07(0.03-0.2) | 0.07(0.02-0.18) |  |
| Marital status |  |  |  |  |  | <0.01 |
| married | 57.9(57.01-58.78) | 54.62(52.86-56.38) | 56.33(54.52-58.12) | 59.74(57.99-61.48) | 61.23(59.46-62.97) |  |
| widowed | 8.15(7.67-8.66) | 6.4(5.54-7.38) | 9.09(8.08-10.2) | 8.63(7.73-9.63) | 8.63(7.66-9.72) |  |
| divorced | 11.45(10.86-12.07) | 12.56(11.32-13.9) | 10.88(9.72-12.15) | 10.8(9.7-12) | 11.5(10.38-12.73) |  |
| never married | 13.58(13.02-14.16) | 17.67(16.49-18.92) | 14.69(13.52-15.94) | 11.54(10.49-12.68) | 10.03(9.04-11.11) |  |
| living with their partners | 6.09(5.71-6.5) | 6.22(5.48-7.04) | 6.04(5.29-6.9) | 6.26(5.48-7.13) | 5.85(5.13-6.68) |  |

Table 1 Continued

| Characters | total | CMIQ1 | CMIQ2 | CMIQ3 | CMIQ4 | P value |
| --- | --- | --- | --- | --- | --- | --- |
| others/not recorded | 0.92(0.76-1.11) | 0.82(0.56-1.19) | 0.96(0.66-1.39) | 0.84(0.56-1.25) | 1.06(0.74-1.5) |  |
| Poverty income ratio |  |  |  |  |  | <0.01 |
| <1.3 | 18.67(18.06-19.28) | 16.03(14.98-17.15) | 18.27(17.05-19.56) | 19.1(17.92-20.34) | 21.49(20.19-22.84) |  |
| 1.3-1.8 | 9.15(8.7-9.62) | 8.1(7.29-8.98) | 9.1(8.22-10.06) | 10.06(9.13-11.08) | 9.44(8.55-10.41) |  |
| >1.8 | 65.1(64.29-65.91) | 68.07(66.52-69.58) | 66.04(64.4-67.65) | 63.8(62.17-65.41) | 62.21(60.52-63.87) |  |
| not recorded | 7.08(6.65-7.54) | 7.8(6.91-8.79) | 6.59(5.78-7.5) | 7.03(6.24-7.92) | 6.86(6.03-7.8) |  |
| Alcohol consumption |  |  |  |  |  | <0.01 |
| non drinking | 10.36(9.87-10.86) | 7.14(6.4-7.96) | 10.61(9.62-11.68) | 10.96(9.97-12.03) | 12.98(11.91-14.13) |  |
| moderate drinking | 32.3(31.44-33.18) | 34.73(33.02-36.48) | 31.55(29.82-33.32) | 32.18(30.48-33.94) | 30.57(28.86-32.33) |  |
| heavy drinking | 41.75(40.86-42.65) | 43.19(41.43-44.97) | 41.93(40.13-43.75) | 41(39.23-42.8) | 40.74(38.96-42.55) |  |
| not recorded | 15.59(15-16.2) | 14.93(13.86-16.08) | 15.92(14.72-17.18) | 15.86(14.71-17.08) | 15.71(14.5-17.01) |  |
| Dyslipidemia |  |  |  |  |  | <0.01 |
| no | 28.93(28.11-29.76) | 59.01(57.27-60.73) | 35.69(33.97-37.45) | 17.07(15.74-18.49) | 1.18(0.85-1.65) |  |
| yes | 71.07(70.24-71.89) | 40.99(39.27-42.73) | 64.31(62.55-66.03) | 82.93(81.51-84.26) | 98.82(98.35-99.15) |  |
| Body Mass Index (kg/m2 ) | 28.78±0.06 | 24.49±0.08 | 27.79±0.1 | 30.29±0.11 | 32.96±0.13 | <0.01 |
| ≤18.5 | 1.6(1.39-1.85) | 4.45(3.78-5.24) | 1.05(0.75-1.45) | 0.51(0.28-0.94) | 0.16(0.06-0.4) |  |
| 18.5-25 | 29.87(29.05-30.71) | 57.79(56.05-59.52) | 33.23(31.52-35) | 17.48(16.19-18.85) | 8.45(7.54-9.47) |  |
| 25-30 | 33.27(32.42-34.12) | 27(25.47-28.58) | 38.23(36.46-40.03) | 37.34(35.61-39.1) | 30.99(29.33-32.69) |  |
| ≥30 | 35.26(34.4-36.12) | 10.75(9.76-11.83) | 27.49(25.94-29.1) | 44.67(42.88-46.47) | 60.4(58.62-62.16) |  |
| Hypertension |  |  |  |  |  | <0.01 |
| no | 52.2(51.3-53.1) | 68.46(66.87-70.01) | 54.94(53.13-56.75) | 46.26(44.47-48.07) | 37.67(35.91-39.46) |  |
| yes | 47.78(46.88-48.68) | 31.54(29.99-33.13) | 45.06(43.25-46.87) | 53.65(51.84-55.45) | 62.33(60.54-64.09) |  |
| not recorded | 0.02(0.01-0.08) | 0(-) | 0(-) | 0.09(0.02-0.32) | 0(-) |  |
| Smoking status |  |  |  |  |  | <0.01 |
| low | 25.5(24.69-26.34) | 27.86(26.24-29.55) | 24.9(23.28-26.6) | 25.68(24.06-27.36) | 23.4(21.83-25.05) |  |
| moderate | 46.88(45.99-47.78) | 47.02(45.26-48.79) | 48.17(46.36-49.99) | 46(44.22-47.78) | 46.3(44.49-48.11) |  |
| high | 27.36(26.55-28.18) | 24.96(23.45-26.54) | 26.69(25.05-28.39) | 28.1(26.5-29.76) | 29.9(28.26-31.6) |  |

Table 1 Continued

| Characters | total | CMIQ1 | CMIQ2 | CMIQ3 | CMIQ4 | P value |
| --- | --- | --- | --- | --- | --- | --- |
| not recorded | 0.25(0.18-0.35) | 0.16(0.07-0.34) | 0.24(0.12-0.47) | 0.22(0.13-0.4) | 0.4(0.24-0.68) |  |
| CVD |  |  |  |  |  | <0.01 |
| no | 91.46(90.98-91.92) | 95.18(94.45-95.83) | 92.21(91.22-93.1) | 91.03(90.06-91.91) | 87.09(85.88-88.22) |  |
| yes | 8.54(8.08-9.02) | 4.82(4.17-5.55) | 7.79(6.9-8.78) | 8.97(8.09-9.94) | 12.91(11.78-14.12) |  |
| cancer |  |  |  |  |  | <0.01 |
| no | 90.52(89.98-91.03) | 91.83(90.8-92.76) | 90.53(89.38-91.57) | 90.21(89.11-91.21) | 89.4(88.24-90.45) |  |
| yes | 9.39(8.88-9.93) | 8.13(7.2-9.17) | 9.45(8.41-10.6) | 9.6(8.62-10.68) | 10.5(9.45-11.65) |  |
| not recorded | 0.09(0.05-0.17) | 0.04(0.01-0.14) | 0.03(0.01-0.09) | 0.19(0.07-0.52) | 0.11(0.03-0.33) |  |
| sedentary behavior |  |  |  |  |  | <0.01 |
| yes | 45.98(45.08-46.87) | 43.9(42.16-45.65) | 46.3(44.49-48.12) | 47.12(45.34-48.92) | 46.76(44.96-48.58) |  |
| no | 53.28(52.38-54.17) | 55.59(53.83-57.33) | 53.12(51.3-54.93) | 52.08(50.28-53.87) | 52.12(50.3-53.93) |  |
| not recorded | 0.75(0.63-0.89) | 0.52(0.35-0.77) | 0.58(0.4-0.84) | 0.8(0.58-1.1) | 1.12(0.83-1.51) |  |
| eGFR | 95.91±0.18 | 100.05±0.36 | 95.95±0.36 | 94.18±0.36 | 93.11±0.37 | <0.01 |
| <15 | 0.15(0.1-0.2) | 0.14(0.06-0.29) | 0.16(0.09-0.28) | 0.17(0.09-0.32) | 0.12(0.06-0.24) |  |
| 15-30 | 0.37(0.29-0.45) | 0.19(0.09-0.4) | 0.24(0.15-0.39) | 0.45(0.31-0.67) | 0.6(0.43-0.83) |  |
| 30-60 | 5.3(4.97-5.66) | 2.97(2.52-3.51) | 5.09(4.44-5.82) | 6.23(5.54-7.01) | 7.12(6.33-7.99) |  |
| 60-90 | 31.6(30.76-32.46) | 27.65(26.06-29.3) | 32.29(30.58-34.05) | 34.05(32.35-35.8) | 32.75(31.06-34.5) |  |
| ≥90 | 62.59(61.71-63.46) | 69.05(67.37-70.68) | 62.22(60.43-63.98) | 59.09(57.31-60.85) | 59.41(57.62-61.18) |  |
| CKD |  |  |  |  |  | <0.01 |
| no | 94.19(93.82-94.53) | 96.7(96.14-97.19) | 94.51(93.76-95.17) | 93.14(92.34-93.87) | 92.16(91.26-92.98) |  |
| yes | 5.81(5.47-6.18) | 3.3(2.81-3.86) | 5.49(4.83-6.24) | 6.86(6.13-7.66) | 7.84(7.02-8.74) |  |
| IR |  |  |  |  |  | <0.01 |
| no | 77.62(76.87-78.35) | 96.47(95.87-96.99) | 86.95(85.67-88.13) | 73.71(72.13-75.22) | 51.56(49.74-53.38) |  |
| yes | 22.38(21.65-23.13) | 3.53(3.01-4.13) | 13.05(11.87-14.33) | 26.29(24.78-27.87) | 48.44(46.62-50.26) |  |
| IFG |  |  |  |  |  | <0.01 |
| no | 79.83(79.1-80.54) | 89.83(88.75-90.82) | 82.64(81.26-83.94) | 76.67(75.13-78.14) | 69.26(67.51-70.96) |  |
| yes | 20.17(19.46-20.9) | 10.17(9.18-11.25) | 17.36(16.06-18.74) | 23.33(21.86-24.87) | 30.74(29.04-32.49) |  |
| T2DM |  |  |  |  |  | <0.01 |
| no | 86.98(86.41-87.53) | 95.61(94.99-96.16) | 90.92(89.98-91.78) | 85.14(83.93-86.27) | 75.46(73.89-76.96) |  |
| yes | 13.02(12.47-13.59) | 4.39(3.84-5.01) | 9.08(8.22-10.02) | 14.86(13.73-16.07) | 24.54(23.04-26.11) |  |

Table 1 Continued

| Characters | total | CMIQ1 | CMIQ2 | CMIQ3 | CMIQ4 | P value |
| --- | --- | --- | --- | --- | --- | --- |
| Labortary parameters |  |  |  |  |  |  |
| Blood urea nitrogen (mmol/L) | 4.85±0.02 | 4.64±0.03 | 4.79±0.03 | 4.91±0.03 | 5.07±0.03 | <0.01 |
| Serum creatinine (µmol/L) | 76.93±0.22 | 73.93±0.44 | 76.87±0.44 | 77.73±0.4 | 79.45±0.48 | <0.01 |
| Total cholesterol (mmol/L) | 4.99±0.01 | 4.76±0.02 | 4.93±0.02 | 5.05±0.02 | 5.23±0.02 | <0.01 |
| Triglyceride (mmol/L) | 1.35±0.01 | 0.68±0 | 1.02±0 | 1.42±0.01 | 2.35±0.01 | <0.01 |
| LDL (mmol/L) | 2.98±0.01 | 2.66±0.01 | 3.01±0.02 | 3.15±0.02 | 3.12±0.02 | <0.01 |
| HDL (mmol/L) | 1.4±0 | 1.79±0.01 | 1.45±0.01 | 1.26±0 | 1.05±0 | <0.01 |
| Body wight (WT, kg) | 82.35±0.2 | 69.4±0.26 | 79.41±0.32 | 86.51±0.36 | 95.27±0.42 | <0.01 |
| Height (HT, cm) | 168.91±0.09 | 168.1±0.17 | 168.9±0.18 | 168.83±0.18 | 169.88±0.19 | <0.01 |
| Waist Circumference (WC, cm) | 98.43±0.15 | 86.19±0.2 | 95.9±0.25 | 102.74±0.26 | 110.01±0.3 | <0.01 |
| HbAlc (%) | 5.57±0.01 | 5.31±0.01 | 5.47±0.01 | 5.62±0.01 | 5.9±0.02 | <0.01 |
| Serum glucose (mmol/L) | 5.49±0.01 | 5.02±0.01 | 5.3±0.02 | 5.57±0.02 | 6.1±0.04 | <0.01 |
| Fasting insulin(FSI, pmol/L) | 74±0.74 | 41.22±0.52 | 59.99±0.88 | 81±1.41 | 116.84±2.25 | <0.01 |
| HOMA-IR | 3.24±0.05 | 1.57±0.02 | 2.46±0.05 | 3.48±0.08 | 5.59±0.15 | <0.01 |

**Note**:Values are weighted mean ± SE or weighted % (95% confidence interval). P values are weighted. Other races include American Indian or Alaska Native, Native Hawaiian or other Pacific Islander, and multiracial persons.

**Abbreviation**: CVD, cardiovascular disease; CKD, Chronic kidney disease; eGFR: estimated glomerular filtration rate; HbAlc, glycosylated hemoglobin; HDL, high density lipoprotein; HOMA-IR, Homeostasis Model Assessment for Insulin Resistance; LDL, low density lipoprotein; NHANES, National Health and Nutrition Examination Survey.

Supplementary Material

# Supplementary Data

Supplementary Material should be uploaded separately on submission. Please include any supplementary data, figures and/or tables.

Supplementary material is not typeset so please ensure that all information is clearly presented, the appropriate caption is included in the file and not in the manuscript, and that the style conforms to the rest of the article.

# Supplementary Figures and Tables

For more information on Supplementary Material and for details on the different file types accepted, please see [here](https://www.frontiersin.org/guidelines/author-guidelines#supplementary-material).

## Supplementary Figures
